# Supplementary material for: Sodium Alginate–Aldehyde Cellulose Nanocrystal Composite Hydrogel for Doxycycline and Other Tetracycline Removal
Source: Nanomaterials (Basel). 2023 Mar 24;13(7):1161. doi: 10.3390/nano13071161 (PMC10096764; doi:10.3390/nano13071161)
Supplement: Supplementary file 1 [file nanomaterials-13-01161-s001.zip › nanomaterials-2261157-supplementary.pdf]

Supporting Information

Sodium Alginate–Aldehyde Cellulose Nanocrystal Composite Hydrogel for Doxycycline and Other Tetracycline Removal

Xiangyu Huang [1], Cheng-Shiuan Lee [2,3], Katherine Zhang [1], Abdulrahman G. Alhamzani [4] and Benjamin S. Hsiao [1,*]

[1] Department of Chemistry, Stony Brook University, 100 Nicolls Road, Stony Brook, NY 11794, USA
[2] New York State Center for Clean Water Technology, Stony Brook University, Stony Brook, NY 11794, USA
[3] Research Center for Environmental Changes, Academia Sinica, Taipei 115, Taiwan
[4] Department of Chemistry, Al-Imam Muhammad Bin Saud Islamic University, Riyadh 11623, Saudi Arabia
* Correspondence: benjamin.hsiao@stonybrook.edu; Tel.: +1-(631)-632-7793

S1. Determination of the aldehyde content in DCNC by titration

An appropriate amount of DCNC suspension (equivalent to 0.1 g in dry weight) was added to 40 mL of 0.25 M hydroxylamine hydrochloride solution (pH = 4.5), where the mixture was stirred at room temperature for 24 h. Subsequently, the released HCl from the Schiff's base reaction was titrated with 0.05 M NaOH until the pH value of the solution returned to 4.5. Titration was also conducted on the same amount of bamboo pulp (pristine_cellulose) in suspension as the reference experiment (blank). The aldehyde content in the mass unit of DCNC was calculated by Equation S1 as follows:

$$\text{Aldehyde content} = C_{NaOH}\,(V_{test} - V_{blank})\,/m \qquad \text{(S1)}$$

where C_{NaOH} is the concentration of the titrating solution (0.05 M); V_{test} and V_{blank} (mL) are the volumes of the NaOH solution consumed in adsorbents or blank sample during titration, respectively, and m (g) is the dry weight of DCNC.
